# The positive association between hemoglobin A1c and the prevalence of periodontitis: A cross-sectional study from NHANES 2009 to 2014

**DOI:** 10.1097/MD.0000000000049755

**Published:** 2026-07-10

**Authors:** Ping Zhang, Xinyue Wang, Yu Jin

**Affiliations:** a Department of Dentistry, Chengdu Integrated TCM and Western Medicine Hospital, Chengdu, Sichuan, China.

**Keywords:** cross-sectional study, hemoglobin A1c (HbA1c), logistic regression, NHANES, periodontitis

## Abstract

Periodontitis is a common chronic inflammatory disease. Hemoglobin A1c (HbA1c) is a key biomarker for long-term glycemic control and has recently been suggested to play a role in the occurrence and progression of periodontitis. However, previous studies have yielded inconclusive results due to limited sample sizes and inconsistent analytical methods. This study aimed to investigate the relationship between HbA1c levels and the prevalence of periodontitis using data from NHANES 2009 to 2014. Periodontitis was defined according to the criteria of the Centers for Disease Control and Prevention and the American Academy of Periodontology. HbA1c was analyzed both as a continuous and categorical variable. Complex survey-weighted logistic regression models were applied to evaluate the associations, and restricted cubic spline regression was used to assess potential dose–response relationships. Subgroup and sensitivity analyses were conducted to further examine the robustness of the findings. A total of 4166 participants were included, with a mean age of 52.0 ± 14.2 years; 2081 were male and 2085 were female. The overall prevalence of periodontitis was 54.7% (2280/4166). Higher HbA1c levels were significantly and positively associated with the prevalence of periodontitis. In the fully adjusted model, each 1% increase in HbA1c was associated with an 18% higher odds of periodontitis (OR = 1.18, 95% CI: 1.03–1.35). Compared with participants with HbA1c < 5.7%, those with HbA1c of 5.7% to 6.4% and ≥ 6.5% had approximately 36% and 75% higher odds, respectively. Restricted cubic spline analysis indicated a linear dose–response relationship between HbA1c and periodontitis. Subgroup analyses showed that the positive association between HbA1c and periodontitis remained significant among males, Mexican Americans, individuals with low income, nondiabetic participants, and those with daily alcohol intake < 10 g/day. Sensitivity analyses confirmed the robustness of the results. Elevated HbA1c levels are significantly and positively associated with the prevalence of periodontitis, suggesting that HbA1c may serve as a potential marker for monitoring periodontal health. Blood glucose control should be considered in the prevention and management of periodontitis, with increased attention to high-risk populations. However, due to the cross-sectional design, causality cannot be inferred from these findings.

## 1. Introduction

Periodontitis is a common chronic inflammatory disease that primarily affects the supporting structures of the teeth, including the gingiva, periodontal ligament, and alveolar bone.^[1,2]^ It is characterized by gingival inflammation, periodontal pocket formation, attachment loss, and alveolar bone resorption, which in severe cases can lead to tooth mobility and even tooth loss.^[3]^ According to the Global Burden of Disease study, periodontitis is one of the most prevalent oral diseases among adults and has a substantial impact on systemic health.^[4,5]^ Evidence suggests that periodontitis is associated with multiple systemic conditions, including diabetes, cardiovascular disease, and metabolic syndrome.^[6–8]^ Therefore, exploring the risk factors related to periodontitis is of great importance for both oral health and systemic health management.

Hemoglobin A1c (HbA1c) is a key biomarker for long-term glycemic control, reflecting the average blood glucose level over the past 2 to 3 months.^[9]^ Although HbA1c is primarily used for the diagnosis and monitoring of diabetes, accumulating evidence indicates that it may also be related to the development and progression of periodontitis.^[10]^ A bidirectional relationship has been proposed between HbA1c and periodontitis. Elevated HbA1c may exacerbate periodontal destruction through impaired immune responses and pro-inflammatory mediators.^[11]^ Conversely, periodontal infection-induced inflammation may, in turn, worsen glycemic control.^[12]^

However, previous studies investigating this relationship have yielded inconclusive results. For instance, some studies were limited by relatively small sample sizes (n < 200),^[11,13]^ which may have reduced statistical power to detect modest associations. Others lacked adequate adjustment for important confounders such as smoking, socioeconomic status, or obesity,^[13]^ or employed varying diagnostic criteria for periodontitis,^[14]^ making cross-study comparisons difficult. More importantly, the shape of the dose–response relationship between HbA1c and periodontitis – whether linear or nonlinear – remains poorly characterized. To address these limitations, this study utilized nationally representative data from the National Health and Nutrition Examination Survey (NHANES) 2009 to 2014 to systematically evaluate the association between HbA1c and periodontitis. We employed multivariable weighted regression to account for complex survey design and restricted cubic spline (RCS) analysis to characterize the dose–response relationship. Furthermore, subgroup and sensitivity analyses were conducted to assess the robustness of the results. Our findings aim to provide further epidemiological evidence on the association between HbA1c and periodontal health and to inform integrated strategies for glycemic control and periodontal prevention.

## 2. Materials and methods

### 2.1. Data source and study population

Data were obtained from NHANES, a program conducted by the National Center for Health Statistics (NCHS), Centers for Disease Control and Prevention (CDC). NHANES is an ongoing, population-based, cross-sectional survey designed to assess the health and nutritional status of the noninstitutionalized United States population. The survey employs a stratified, multistage, probability sampling design to ensure nationally representative estimates. NHANES data include demographic information, lifestyle factors, laboratory tests, anthropometric measurements, and clinical health evaluations, along with comprehensive periodontal examination data. All data are collected using standardized protocols established by NCHS, with strict quality control and validation procedures to ensure reliability and comparability. Because NHANES data are publicly available and de-identified, this study complied with NCHS ethical regulations, and no additional institutional review board approval was required.^[15]^ The study population was drawn from participants in NHANES 2009 to 2014. To ensure accuracy and comparability, we applied the following exclusion criteria: participants without periodontal examination data, those missing HbA1c measurements, and those lacking covariate information.

### 2.2. Periodontitis diagnosis

Periodontitis was defined according to the joint case definition proposed by the CDC and the American Academy of Periodontology.^[16,17]^ Periodontal examinations measured clinical attachment loss (CAL) and probing pocket depth (PPD) at 6 sites per tooth for up to 28 teeth (168 sites) per participant. Mild periodontitis was defined as the presence of at least 2 interproximal sites (on different teeth) with CAL ≥ 3 mm and at least 2 interproximal sites with PPD ≥ 4 mm, or at least 1 site with PPD ≥ 5 mm. Moderate periodontitis was defined as at least 2 interproximal sites (on different teeth) with CAL ≥ 4 mm, or at least 2 interproximal sites with PPD ≥ 5 mm. Severe periodontitis was defined as at least 2 interproximal sites (on different teeth) with CAL ≥ 6 mm and at least 1 interproximal site with PPD ≥ 5 mm. Participants who did not meet any of the above criteria were classified as having no periodontitis.

### 2.3. HbA1c measurement

HbA1c levels were obtained from NHANES 2009 to 2014 data and measured using high-performance liquid chromatography. Blood samples were collected by trained personnel through venipuncture and analyzed in CDC-certified laboratories, with assays performed at the University of Minnesota Medical Center laboratory during 2009 to 2012 and at the University of Missouri–Columbia laboratory during 2013 to 2014. HbA1c results were expressed as percentages (%) and categorized according to the American Diabetes Association (ADA) criteria into normal glycemia (<5.7%), prediabetes (5.7%–6.4%), and diabetes (≥6.5%).^[18]^ In this study, HbA1c was analyzed both as a continuous variable and as a categorical variable based on these thresholds. The continuous analysis assessed the dose–response relationship per unit increase in HbA1c, while the categorical analysis facilitated clinical interpretation using established glycemic categories.

### 2.4. Covariates

Covariates were selected based on prior literature and theoretical considerations as potential confounders in the association between HbA1c and periodontitis. Several potential confounders were included as covariates to control for possible confounding effects in the association between HbA1c and periodontitis. Demographic characteristics comprised age, gender, race/ethnicity (Mexican American, Other Hispanic, Non-Hispanic White, Non-Hispanic Black, and Other Race), education level (less than high school, high school or equivalent, and college or above), marital status (married, widowed, divorced, separated, never married, and living with partner),^[19]^ and socioeconomic status assessed using the poverty income ratio (PIR). Health status and lifestyle factors included body mass index (BMI), diabetes (yes/no), daily alcohol intake (<10 g/day and ≥ 10 g/day),^[20]^ daily energy intake, smoking status (never smoking, former smoking, and current smoking),^[21]^ and hypertension (yes/no). Biochemical indicators comprised uric acid, total cholesterol, triglycerides, and serum insulin.

### 2.5. Statistical analyses

Considering the complex sampling design of NHANES, weighted analyses were performed to ensure nationally representative estimates. The clustering, stratification, and sampling weights provided by NCHS were incorporated into all analyses. HbA1c was analyzed both as a continuous variable and as a categorical variable according to the ADA criteria (normal glycemia: <5.7%; prediabetes: 5.7%–6.4%; diabetes: ≥6.5%), while periodontitis was treated as a binary outcome (no/yes). For baseline characteristics, continuous variables were presented as means ± standard deviations or medians with interquartile ranges, depending on distribution, and group differences were tested using 1-way ANOVA or the Kruskal–Wallis test. Categorical variables were expressed as frequencies (percentages) and compared using the χ^2^ test. All estimates in Table 1 are presented as unweighted values to describe the analytic sample. Univariate associations between HbA1c and periodontitis were assessed using weighted logistic regression, with results expressed as odds ratios and 95% confidence intervals. Multivariable logistic regression was then conducted with progressive adjustment for covariates across 4 models: Model 1, unadjusted; Model 2, adjusted for age, gender, education level, marital status, race/ethnicity, and PIR; Model 3, further adjusted for BMI, alcohol intake, energy intake, smoking status, hypertension, uric acid, total cholesterol, and triglycerides; and Model 4, further adjusted for diabetes status and serum insulin. Multicollinearity among covariates was assessed using variance inflation factors. RCS analyses were used to explore potential nonlinear dose–response relationships between HbA1c and periodontitis. Subgroup analyses were stratified by various demographic and clinical characteristics, and heterogeneity was evaluated by including interaction terms, with *P* < .05 considered significant. Sensitivity analyses were performed in 2 ways to assess the robustness of our findings. First, to address potential over-adjustment due to including diabetes status and serum insulin in the fully adjusted model, we repeated the primary analyses restricted to participants without diagnosed diabetes. Second, to evaluate the impact of missing covariate data, we conducted simple imputation for missing covariates (using the mean for normally distributed variables, the median for non-normally distributed variables, and the mode for categorical variables) to generate a complete dataset and re-ran the main analyses to test robustness. All analyses were performed using R software (The R Foundation, http://www.R-project.org) and Free Statistics software (version 1.9). A 2-sided α level of 0.05 was considered statistically significant.

**Table 1:** Baseline characteristics of study participants according to HbA1c categories.

		HbA1c < 5.7%	HbA1c 5.7–6.4%	HbA1c ≥ 6.5%	
Variables	Total (n = 4166)	(n = 2387)	(n = 1301)	(n = 478)	*P*-value
Periodontitis, n (%)					
No	1886 (45.3)	1292 (54.1)	456 (35)	138 (28.9)	<.001
Yes	2280 (54.7)	1095 (45.9)	845 (65)	340 (71.1)	
Age (years), Mean ± SD	52.0 ± 14.2	48.2 ± 13.5	56.8 ± 13.7	58.1 ± 12.6	<.001
Gender, n (%)					
Male	2081 (50.0)	1179 (49.4)	641 (49.3)	261 (54.6)	.214
Female	2085 (50.0)	1208 (50.6)	660 (50.7)	217 (45.4)	
Race/ethnicity, n (%)					
Mexican American	587 (14.1)	307 (12.9)	201 (15.4)	79 (16.5)	<.001
Other Hispanic	394 (9.5)	213 (8.9)	132 (10.1)	49 (10.3)	
Non-Hispanic White	1938 (46.5)	1263 (52.9)	498 (38.3)	177 (37)	
Non-Hispanic Black	788 (18.9)	335 (14)	330 (25.4)	123 (25.7)	
Other Race	459 (11.0)	269 (11.3)	140 (10.8)	50 (10.5)	
Education, n (%)					
Less than high school	921 (22.1)	434 (18.2)	342 (26.3)	145 (30.3)	<.001
High school or equivalent	887 (21.3)	471 (19.7)	305 (23.4)	111 (23.2)	
College or above	2358 (56.6)	1482 (62.1)	654 (50.3)	222 (46.4)	
Marital status, n (%)					
Married	2469 (59.3)	1433 (60)	759 (58.3)	277 (57.9)	<.001
Widowed	283 (6.8)	120 (5)	117 (9)	46 (9.6)	
Divorced	519 (12.5)	294 (12.3)	156 (12)	69 (14.4)	
Separated	144 (3.5)	87 (3.6)	44 (3.4)	13 (2.7)	
Never married	464 (11.1)	282 (11.8)	131 (10.1)	51 (10.7)	
Living with partner	287 (6.9)	171 (7.2)	94 (7.2)	22 (4.6)	
PIR, Median (IQR)	2.3 (1.1, 4.3)	2.6 (1.2, 4.6)	2.1 (1.1, 4.1)	2.0 (1.1, 3.5)	<.001
BMI (kg/m^2^), Mean ± SD	29.4 ± 6.8	28.1 ± 6.2	30.3 ± 6.8	33.2 ± 7.6	<.001
Diabetes, n (%)					
No	3654 (87.7)	2352 (98.5)	1165 (89.5)	137 (28.7)	<.001
Yes	512 (12.3)	35 (1.5)	136 (10.5)	341 (71.3)	
Daily alcohol intake, n (%)					
<10 g/day	3261 (78.3)	1772 (74.2)	1076 (82.7)	413 (86.4)	<.001
≥10 g/day	905 (21.7)	615 (25.8)	225 (17.3)	65 (13.6)	
Smoking status, n (%)					
Never smoker	2343 (56.2)	1386 (58.1)	694 (53.3)	263 (55)	.183
Former smoker	1066 (25.6)	565 (23.7)	359 (27.6)	142 (29.7)	
Current smoker	757 (18.2)	436 (18.3)	248 (19.1)	73 (15.3)	
Hypertension, n (%)					
No	2309 (55.4)	1581 (66.2)	581 (44.7)	147 (30.8)	<.001
Yes	1857 (44.6)	806 (33.8)	720 (55.3)	331 (69.2)	
Daily energy intake (kcal), Mean ± SD	2068.9 ± 845.5	2115.9 ± 861.6	2011.3 ± 773.4	1991.2 ± 934.1	< 0.001
Uric acid (mg/dL), Mean ± SD	5.5 ± 1.4	5.3 ± 1.4	5.7 ± 1.4	5.8 ± 1.5	<.001
Total cholesterol (mg/dL), Mean ± SD	196.8 ± 41.1	196.8 ± 38.9	199.9 ± 42.0	188.7 ± 47.9	.005
Triglycerides (mg/dL), Median (IQR)	104.0 (74.0, 154.0)	96.0 (68.0, 141.0)	110.0 (80.0, 162.0)	134.5 (90.0, 206.5)	<.001
Serum insulin (µU/mL), Median (IQR)	10.3 (6.4, 16.8)	8.9 (5.9, 13.7)	12.2 (7.4, 19.1)	14.9 (9.2, 23.9)	<.001

HbA1c = hemoglobin A1c, PIR = poverty income ratio.

## 3. Results

### 3.1. Study population selection flowchart

**Figure 1.**
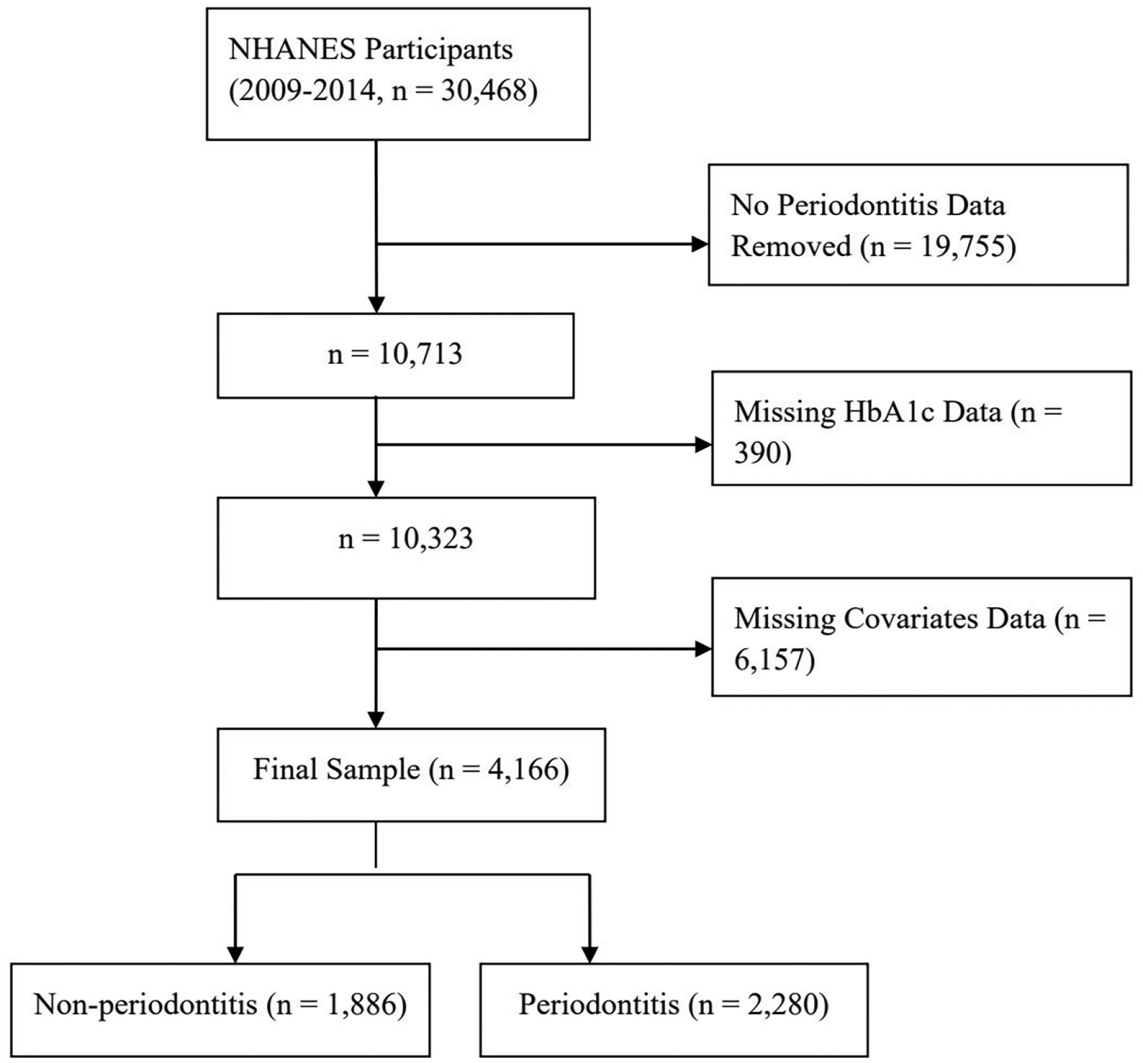
Flowchart of study population selection from NHANES 2009 to 2014. NHANES = National Health and Nutrition Examination Survey.

This study was based on NHANES 2009 to 2014 data, including a total of 30,468 participants. First, individuals without periodontal examination data were excluded (n = 19,755), leaving 10,713 participants. Second, individuals without HbA1c data were excluded (n = 390), resulting in 10,323 participants. Third, individuals with missing covariate data were excluded (n = 6157), leaving a final sample of 4166 participants for analysis. Among them, 1886 (45.3%) were free of periodontitis, while 2280 (54.7%) were diagnosed with periodontitis (Fig. 1).

### 3.2. Baseline characteristics of the study population

A total of 4166 participants aged over 30 years were included in this study, all of whom were aged 30 years or older (mean age 52.0 ± 14.2 years). The cohort consisted of 2081 males and 2085 females. Based on HbA1c levels, participants were categorized into the normoglycemia group (HbA1c < 5.7%, n = 2387), the prediabetes group (HbA1c 5.7%–6.4%, n = 1301), and the diabetes group (HbA1c ≥ 6.5%, n = 478). The overall prevalence of periodontitis was 54.7% (n = 2280). Among the subgroups, the prevalence was highest in the diabetes group (71.1%), significantly higher than in the prediabetes group (65.0%) and the normoglycemia group (45.9%) (*P* < .001). In the HbA1c ≥ 6.5% group, participants exhibited a markedly higher burden of disease and metabolic abnormalities. Participants in the HbA1c ≥ 6.5% group were older on average, with a relatively higher proportion of non-Hispanic Blacks, an increased proportion of individuals with less than a high school education, and a higher proportion of widowed individuals. Their socioeconomic status was poorer, as indicated by a lower PIR. In terms of physical and metabolic characteristics, this group had a significantly higher BMI and the highest prevalence of both diabetes and hypertension. Regarding lifestyle factors, alcohol consumption was lower and daily energy intake was the lowest among all groups. Biochemical indicators showed elevated uric acid, triglycerides, and serum insulin, whereas total cholesterol levels were the lowest.

### 3.3. Association between HbA1c and the prevalence of periodontitis

Table 2 presents the association between HbA1c levels and the prevalence of periodontitis. VIF analysis showed no evidence of problematic multicollinearity among the covariates (all variance inflation factors < 2; mean VIF = 1.28; Supplementary Table 1, Supplemental Digital Content 1). When HbA1c was analyzed as a continuous variable, each 1% increase in HbA1c was associated with 18% higher odds of periodontitis in the fully adjusted model (Model 4) (OR = 1.18, 95% CI: 1.03–1.35, *P* = .021). When HbA1c was categorized, compared with the HbA1c < 5.7% group, the odds of periodontitis were consistently higher in both the 5.7%–6.4% group and the HbA1c ≥ 6.5% group across all 4 models. Trend analyses across all 4 models consistently indicated a significant positive association between HbA1c levels and the odds of periodontitis (P for trend < 0.001, 0.004, 0.002, and 0.004, respectively).

**Table 2:** The associations between HbA1c and the prevalence of periodontitis.

	Model 1		Model 2		Model 3		Model 4	
	OR (95%CI)	P value	OR (95%CI)	P value	OR (95%CI)	*P*-value	OR (95%CI)	*P*-value
Continuous	1.51 (1.33–1.72)	<0.001	1.17 (1.06–1.30)	0.004	1.20 (1.07–1.36)	0.004	1.18 (1.03–1.35)	0.021
Categories								
HbA1c < 5.7%	Reference		Reference		Reference		Reference	
5.7% ≤ HbA1c < 6.4%	2.23 (1.82–2.73)	<0.001	1.38 (1.10–1.72)	0.006	1.37 (1.10–1.71)	0.007	1.36 (1.09–1.69)	0.008
HbA1c ≥ 6.5%	3.05 (2.24–4.15)	<0.001	1.63 (1.13–2.36)	0.011	1.83 (1.23–2.74)	0.005	1.75 (1.11–2.75)	0.018
*P* for trend	–	<0.001	–	0.004	–	0.002		0.004

Model 1: Unadjusted.

Model 2: Adjusted for demographic factors: age, gender, race/ethnicity, education level, marital status, and PIR.

Model 3: Additionally adjusted for BMI, daily alcohol intake, smoking status, hypertension, daily energy intake, uric acid, total cholesterol, and triglycerides.

Model 4: Further adjusted for diabetes status and serum insulin.

HbA1c = hemoglobin A1c, PIR = poverty income ratio.

### 3.4. RCS analysis results

**Figure 2.**
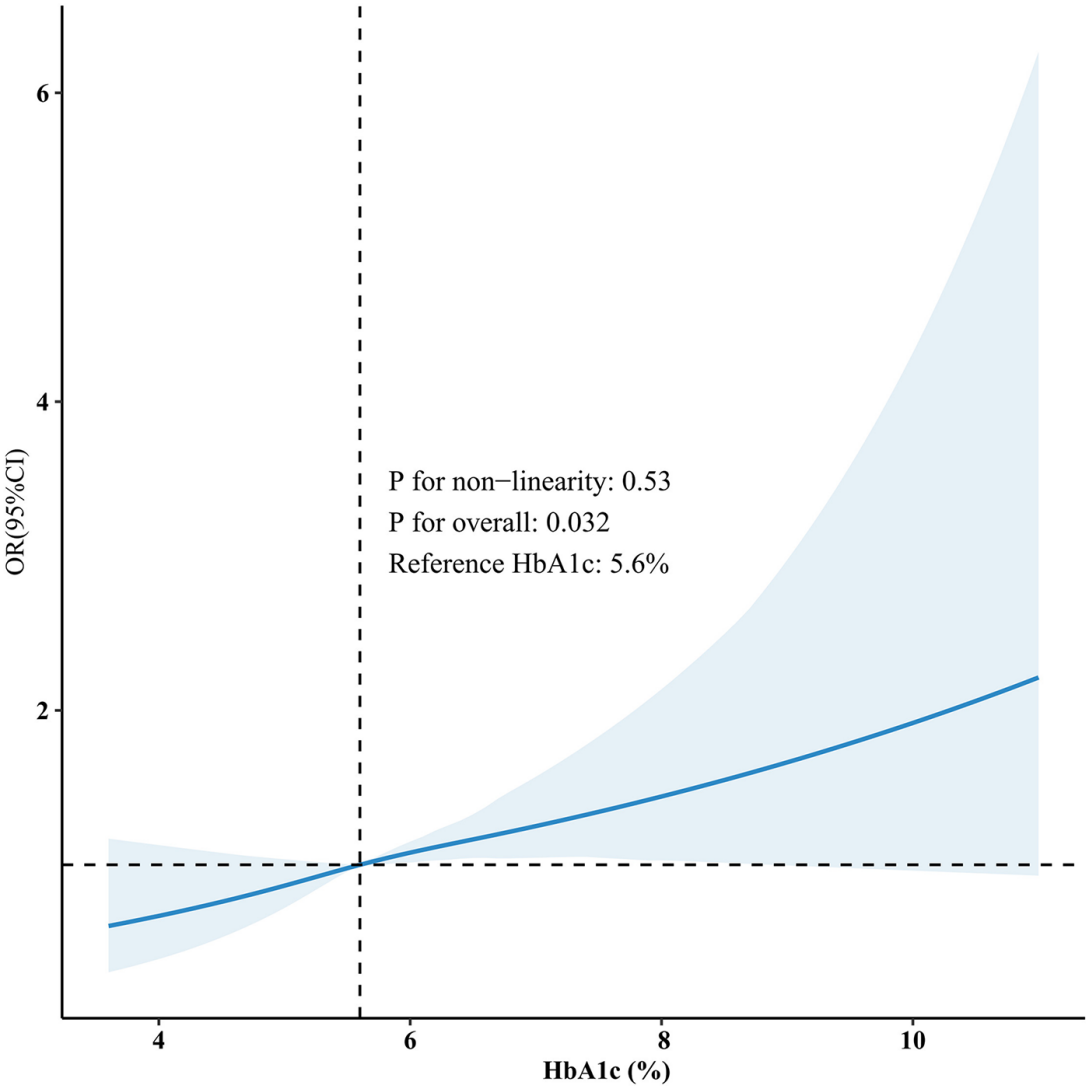
RCS analysis of the association between HbA1c levels and periodontitis risk. RCS = restricted cubic spline.

RCS analysis revealed a significant positive association between HbA1c levels and the risk of periodontitis (P for overall = 0.032). As HbA1c increased, the risk of periodontitis gradually rose. The test for nonlinearity was not significant (P for nonlinearity = 0.53), suggesting that the relationship between HbA1c and periodontitis was primarily linear (Fig. 2). The widening confidence intervals at higher HbA1c levels should be noted, likely reflecting the smaller sample size in this range.

### 3.5. Subgroup analyses and interaction effects

**Figure 3.**
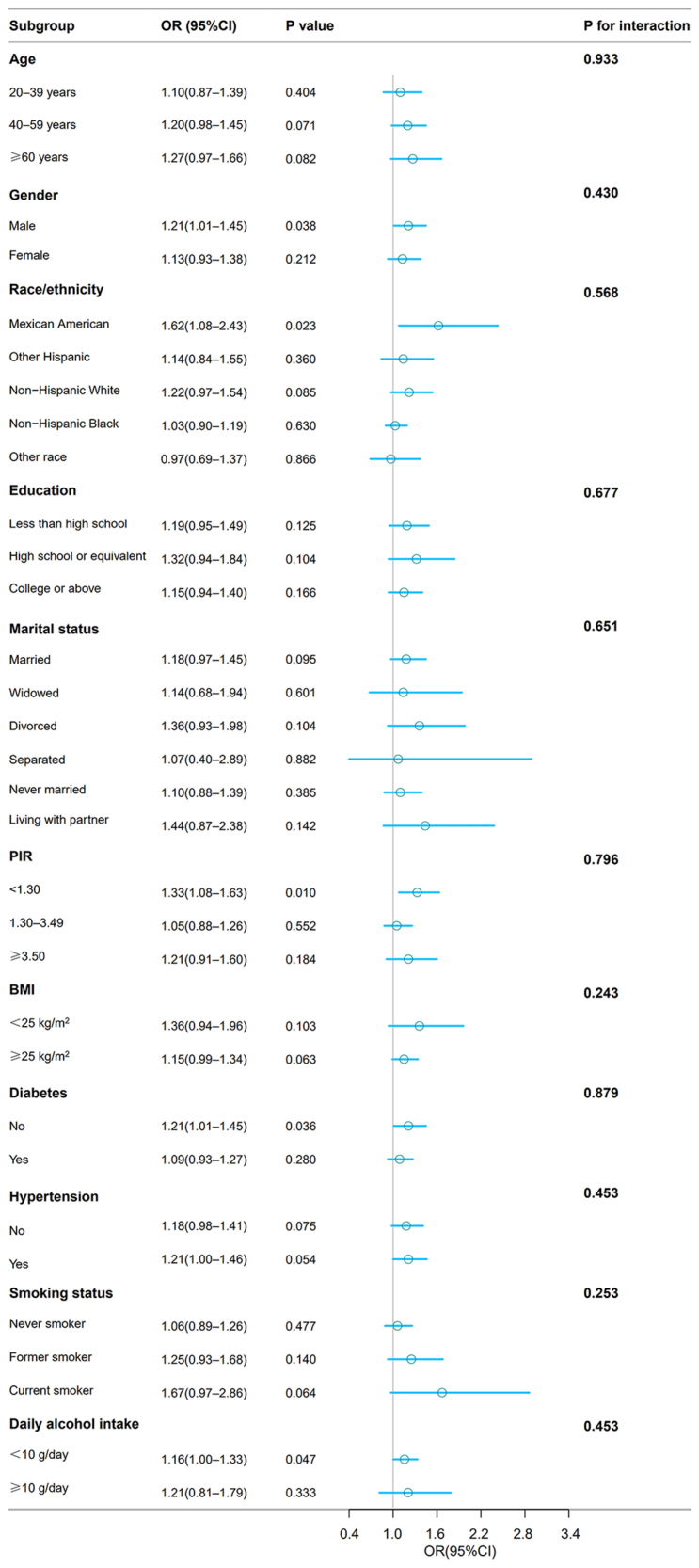
Subgroup analyses of the association between HbA1c levels and periodontitis.

The overall trend demonstrated a positive association between elevated HbA1c levels and the odds of periodontitis. This association reached statistical significance in several subgroups (*P* < .05): males (OR = 1.21, 95% CI: 1.01–1.45, *P* = .038), Mexican Americans (OR = 1.62, 95% CI: 1.08–2.43, *P* = .023), individuals with lower socioeconomic status (PIR < 1.30) (OR = 1.33, 95% CI: 1.08–1.63, *P* = .010), participants without diabetes (OR = 1.21, 95% CI: 1.01–1.45, *P* = .036), and those with a daily alcohol intake of <10 g/day (OR = 1.16, 95% CI: 1.00–1.33, *P* = .047). However, interaction analyses did not reveal any statistically significant effect modification across subgroups (all P for interaction > 0.05). Detailed results are presented in Figure 3.

### 3.6. Sensitivity analysis results

To assess the robustness of our findings, we conducted a sensitivity analysis among participants without a diagnosed history of diabetes to eliminate the potential confounding effect of known diabetes on the association between HbA1c and periodontitis (Table 3). When HbA1c was treated as a continuous variable, each 1% increase in HbA1c was associated with a 21% higher risk of periodontitis in the fully adjusted model (Model 4) (OR = 1.21, 95% CI: 1.01–1.45, *P* = .036). After grouping HbA1c into quartiles, participants in the highest quartile (Q4: HbA1c = 6.2% ± 0.9%) had a significantly higher risk of periodontitis compared to those in the lowest quartile (Q1: HbA1c = 5.0% ± 0.2%) in the fully adjusted model (OR = 1.43, 95% CI: 1.05–1.97, *P* = .027), with a significant trend across quartiles (P for trend = 0.037). To address missing data, imputation was performed for variables with missing values, and the analysis was repeated using the completed dataset (Table 4). The results remained robust: in the fully adjusted model (Model 4), each 1% increase in HbA1c was associated with a 14% higher risk of periodontitis (OR = 1.14, 95% CI: 1.05–1.23, *P* = .003). Compared with participants with HbA1c < 5.7%, those with HbA1c levels of 5.7–6.4% and ≥ 6.5% had a 21% (OR = 1.21, 95% CI: 1.03–1.41, *P* = .019) and 35% (OR = 1.35, 95% CI: 0.98–1.85, *P* = .065) increased risk of periodontitis, respectively, showing a significant dose–response trend (P for trend = 0.013).

**Table 3:** Sensitivity analysis: the associations between HbA1c and the prevalence of periodontitis among undiagnosed diabetes participants.

	Model 1		Model 2		Model 3		Model 4	
	OR (95% CI)	*P*-value	OR (95%CI)	*P*-value	OR (95%CI)	P value	OR (95% CI)	*P*-value
Continuous	1.90 (1.46–2.46)	<.001	1.20 (1.03–1.39)	.022	1.23 (1.03–1.47)	0.025	1.21 (1.01–1.45)	.036
Quartile (Mean ± SD, %)								
Q1 (5.0 ± 0.2)	Reference	–	Reference	–	Reference	–	Reference	–
Q2 (5.4 ± 0.1)	1.34 (1.02–1.77)	.036	1.08 (0.80–1.46)	.606	1.05 (0.77–1.42)	0.750	1.04 (0.77–1.41)	.782
Q3 (5.6 ± 0.1)	1.73 (1.45–2.08)	<.001	1.07 (0.85–1.34)	.569	1.07 (0.84–1.36)	0.585	1.06 (0.83–1.35)	.624
Q4 (6.2 ± 0.9)	2.96 (2.39–3.67)	<.001	1.44 (1.10–1.89)	.009	1.47 (1.07–2.03)	0.020	1.43 (1.05–1.97)	.027
*P* for trend	–	<.001	–	.015	–	0.026	–	.037

Model 1: Unadjusted.

Model 2: Adjusted for demographic factors: age, gender, race/ethnicity, education level, marital status, and PIR.

Model 3: Additionally adjusted for BMI, daily alcohol intake, smoking status, hypertension, daily energy intake, uric acid, total cholesterol, and triglycerides.

Model 4: Further adjusted for serum insulin.

HbA1c = hemoglobin A1c, PIR = poverty income ratio.

**Table 4:** The associations between HbA1c and the prevalence of periodontitis (sensitivity analyses after simple imputation for missing values).

	Model 1		Model 2		Model 3		Model 4	
	OR (95%CI)	*P*-value	OR (95%CI)	*P*-value	OR (95%CI)	P value	OR (95%CI)	*P*-value
Continuous	1.51 (1.39–1.64)	<.001	1.15 (1.08–1.22)	<.001	1.14 (1.07–1.21)	<0.001	1.14 (1.05–1.23)	.003
Categories								
HbA1c < 5.7%	Reference	–	Reference	–	Reference	–	Reference	–
5.7% ≤ HbA1c < 6.4%	2.08 (1.82–2.37)	<.001	1.27 (1.08–1.49)	.005	1.21 (1.04–1.41)	0.015	1.21 (1.03–1.41)	.019
HbA1c ≥ 6.5%	2.75 (2.23–3.39)	<.001	1.42 (1.14–1.77)	.003	1.40 (1.11–1.77)	0.007	1.35 (0.98–1.85)	.065
*P* for trend	–	<.001	–	<.001	–	0.003	–	.013

Model 1: Unadjusted.

Model 2: Adjusted for demographic factors: age, gender, race/ethnicity, education level, marital status, and PIR.

Model 3: Additionally adjusted for BMI, daily alcohol intake, smoking status, hypertension, daily energy intake, uric acid, total cholesterol, and triglycerides.

Model 4: Further adjusted for diabetes status and serum insulin.

HbA1c = hemoglobin A1c, PIR = poverty income ratio.

## 4. Discussion

This study, based on nationally representative data from the NHANES 2009 to 2014 cycles, investigated the association between HbA1c levels and the prevalence of periodontitis. The results demonstrated a significant positive association between elevated HbA1c levels and the risk of periodontitis, which remained robust even after adjusting for multiple confounding factors. Further dose–response analysis using RCS suggested a linear positive relationship between HbA1c and periodontitis. In subgroup analyses, the association was more pronounced among males, Mexican Americans, individuals with low income, participants without diabetes, and those with a daily alcohol intake of <10 g/day, indicating that the impact of HbA1c on periodontal health may vary across different population groups. These findings provide additional evidence supporting the potential role of HbA1c as a risk factor for periodontitis and offer new insights for future disease prevention strategies and clinical management.

Our findings are consistent with previous reports. Previous studies have also indicated a positive association between elevated HbA1c levels and an increased risk of periodontitis. Morita et al conducted a longitudinal study involving 5856 participants with baseline periodontal pockets < 4 mm and found that individuals with baseline HbA1c ≥ 6.5% had a significantly higher risk of developing periodontal pockets ≥ 4 mm (CPI score 3 or 4) over a 5-year follow-up period. After adjusting for BMI, smoking status, gender, and age, the relative risk was 1.17 (*P* = .038), suggesting that elevated HbA1c levels are associated with an increased risk of periodontitis.^[22]^ Zhao et al., through a systematic review including case–control and cross-sectional studies, reported that among nondiabetic populations, individuals with periodontitis had significantly higher HbA1c levels compared to periodontally healthy individuals. They recommended HbA1c screening in nondiabetic patients with periodontitis to identify potential hyperglycemia.^[10]^ Musurlieva et al., in a case–control study involving 228 Bulgarian patients with chronic periodontitis, identified type 2 diabetes mellitus as one of the main factors closely associated with the progression of periodontitis.^[23]^ On the other hand, periodontitis may also influence HbA1c levels. Costa et al included 80 patients with type 2 diabetes and found that the progression of periodontitis was associated with increased HbA1c levels.^[24]^ Similarly, a study by Kocher et al demonstrated that nonsurgical periodontal treatment significantly improved glycemic control in patients with both prediabetes and periodontitis, with HbA1c levels decreasing from 5.9% to 5.4% after 15.5 months of follow-up.^[25]^

Compared with previous studies, the present study has several notable strengths. First, it is based on nationally representative data from NHANES, which includes a diverse population and a wide range of health indicators, thereby enhancing the external validity of the findings. Second, the sample size in this study was relatively large, providing greater statistical power compared to many single-center studies and contributing to the robustness of the results. Third, we employed multivariable regression models incorporating a wide array of potential confounders, including demographic characteristics, lifestyle factors, and biochemical indicators, which helped to minimize residual confounding. Additionally, we used RCS analysis to characterize the dose–response relationship between HbA1c levels and periodontitis, which provided a more nuanced understanding of this association. We also conducted extensive subgroup and sensitivity analyses to assess the robustness of our findings. In the sensitivity analyses, consistent results were observed after excluding participants with diabetes and after imputation for missing data. However, it is important to emphasize that due to the cross-sectional design, temporality cannot be established, and causal inferences cannot be drawn from these findings. Collectively, these strengths provide further epidemiological evidence to inform future population-level risk stratification and targeted intervention strategies.

The observed association may be explained by multiple biological mechanisms. Elevated HbA1c levels may be associated with the onset and progression of periodontitis through multiple mechanisms. First, increased HbA1c promotes the production of pro-inflammatory cytokines such as IL-1β, IL-6, and TNF-α, which sustain chronic inflammation in periodontal tissues, leading to alveolar bone resorption and tissue destruction.^[26–29]^ Second, elevated HbA1c may alter the oral microbiota, facilitating the colonization and growth of periodontal pathogens. This microbial imbalance can further trigger host inflammatory responses and aggravate periodontal damage.^[30,31]^ High HbA1c levels may impair host immune defense mechanisms and induce microvascular complications, thereby reducing the regenerative capacity of periodontal tissues and increasing the risk of tissue destruction and bone loss.^[32,33]^ It is worth noting that the relationship between HbA1c and periodontitis is bidirectional. Studies have shown that chronic inflammation caused by periodontitis can increase insulin resistance, thereby impairing glycemic control.^[34,35]^ Conversely, systematic reviews and clinical trials have demonstrated that periodontal treatment – such as scaling and root planing – can lead to reductions in HbA1c levels,^[25,36]^ suggesting that improving periodontal health may contribute to better glycemic management in patients with diabetes.

The findings of this study have important implications for clinical practice. First, elevated HbA1c levels may serve as a potential predictive marker for periodontitis risk. Therefore, glycemic monitoring should be considered as part of periodontal health assessments, particularly in high-risk populations such as individuals with prediabetes or those with low socioeconomic status. Second, the management of periodontitis should not be limited to local interventions but should also take systemic metabolic conditions – especially glycemic control – into account. For individuals with elevated HbA1c levels, a comprehensive periodontal care approach may help reduce the risk of periodontal disease while simultaneously improving glycemic outcomes. Moreover, given the bidirectional relationship between HbA1c and periodontitis, screening and intervention strategies for periodontal disease should be closely integrated into diabetes management. Interdisciplinary collaboration between periodontists and endocrinologists should be encouraged to optimize overall health outcomes. Future longitudinal studies and randomized controlled trials are warranted to evaluate the long-term impact of personalized periodontal health management strategies on systemic health.

While our findings are promising, several limitations should be acknowledged. First, due to its cross-sectional design, this study cannot establish a causal relationship between HbA1c levels and periodontitis; longitudinal studies are needed to confirm this association. Second, the exclusion of participants with missing covariate data (n = 6157) may introduce selection bias. However, although simple imputation yielded broadly consistent results, this method does not fully account for uncertainty associated with missing data and, therefore, may not completely eliminate potential bias. Given the extent of missing covariate data and exclusions, the possibility of selection bias cannot be entirely ruled out. Future studies should consider using more robust methods, such as multiple imputation, to better address missingness. Third, no formal power calculation was performed a priori; however, the large sample size and the statistically significant associations observed suggest adequate power to detect clinically meaningful effects. Fourth, because diabetes status and serum insulin are closely related to HbA1c, including these variables in the fully adjusted model may have introduced potential over-adjustment. However, an additional model excluding these 2 variables yielded materially similar results, suggesting that the main findings were not substantially affected. However, sensitivity analyses restricted to nondiabetic participants yielded consistent results, suggesting that any potential over-adjustment did not materially bias our findings. Fifth, NHANES data are primarily based on physical examinations and self-reported questionnaires, which may introduce information bias. For example, the diagnosis of periodontitis was based on partial-mouth examinations, which may have led to an underestimation of the actual prevalence. HbA1c reflects average blood glucose levels over the past 2 to 3 months and may not fully capture long-term glycemic control. Furthermore, although multiple potential confounders were adjusted for, residual confounding due to unmeasured variables cannot be entirely ruled out. Future research should account for these factors and validate the robustness of the findings through prospective cohort studies or interventional trials.

## 5. Conclusion

Based on data from NHANES 2009 to 2014, this study found a significant association between elevated HbA1c levels and an increased odds of periodontitis, which remained robust after adjustment for multiple confounding factors and was more pronounced in specific subpopulations. These findings suggest that HbA1c may serve as a potential predictive marker for periodontitis, offering new insights for early screening and intervention strategies. Future longitudinal studies are needed to confirm the causal relationship and to explore personalized prevention and intervention approaches aimed at improving both systemic and oral health.

## Acknowledgments

We would like to thank the DeepL translation tool for its assistance in language editing, which improved the overall readability of the manuscript. All authors independently reviewed the final version to ensure the accuracy and integrity of the original content were maintained.

## Author contributions

**Conceptualization:** Ping Zhang, Xinyue Wang, Yu Jin.

**Data curation:** Ping Zhang, Xinyue Wang.

**Formal analysis:** Ping Zhang.

**Methodology:** Ping Zhang, Yu Jin.

**Software:** Ping Zhang, Xinyue Wang.

**Supervision:** Yu Jin.

**Validation:** Xinyue Wang, Yu Jin.

**Visualization:** Ping Zhang, Xinyue Wang.

**Writing – original draft:** Ping Zhang, Xinyue Wang, Yu Jin.

**Writing – review & editing:** Ping Zhang, Xinyue Wang, Yu Jin.


