# Supplementary material for: The positive association between hemoglobin A1c and the prevalence of periodontitis: A cross-sectional study from NHANES 2009 to 2014
Source: Medicine (Baltimore). 2026 Jul 10;105(28):e49755. doi: 10.1097/MD.0000000000049755 (PMC13363198; doi:10.1097/MD.0000000000049755)
Supplement: Supplementary file 1 [file medi-105-e49755-s001.docx]

**Supplementary Table 1.** VIF analysis of covariates included in the multivariable logistic regression model.

| **Variables** | **VIF** |
| --- | --- |
| Gender | 1.51 |
| Uric acid | 1.47 |
| Age | 1.36 |
| BMI | 1.36 |
| PIR | 1.36 |
| Education level | 1.34 |
| Daily energy intake | 1.33 |
| Hypertension | 1.33 |
| Triglycerides | 1.29 |
| Total cholesterol | 1.24 |
| Serum insulin | 1.21 |
| Daily alcohol intake | 1.17 |
| Diabetes status | 1.14 |
| Race/ethnicity | 1.13 |
| Marital status | 1.13 |
| Smoking status | 1.11 |
